# Medical Schools

**Published:** 1845-08-01

**Authors:** 


*Medical Schools.*—We have received Catalogues and Circulars from the Medical Department of the Western Reserve College located at Cleveland; from the Medical Department of Harvard University, Boston; and from the Medical College of the state of S. Carolina, Charleston.

By the first we perceive that a new college edifice is being erected at Cleveland, and that much advantage is expected from the Marine Hospital now in progress of erection in that place. The Boston School, it appears, has doubled the number of its students within the last five years. The number of the last class was 157. It is also stated in their circular that a "greater number of teachers for the different medical schools of the United States, in proportion, have been taken from their graduates, than from any other institution in the country." The Charleston School appears to be in a flourishing condition. Their last class numbered 196. No. of graduates 94.

